# Number 2 Feibi Recipe Reduces PM2.5-Induced Lung Injury in Rats

**DOI:** 10.1155/2018/3674145

**Published:** 2018-01-09

**Authors:** Zhaoheng Liu, Wei Wang, Fang Cao, Shuo Liu, Xinxin Zou, Guodong Li, Haojie Yang, Yang Jiao

**Affiliations:** ^1^Beijing University of Chinese Medicine, No. 11 Bei San Huan Dong Lu, Chaoyang District, Beijing 100029, China; ^2^Respiratory Department, Chongqing Traditional Chinese Medicine Hospital in Dao Men Kou, No. 40 Dao Men Kou, Yuzhong District, Chongqing 400011, China; ^3^Dongfang Hospital Affiliated to Beijing University of Chinese Medicine, No. 6, Fang Zhuang, Fang Xing Yuan, Fengtai District, Beijing 100078, China

## Abstract

Air pollution is the main cause of respiratory diseases. Fine particulates with the diameter below 2.5 *μ*m can get into the alveoli and then enter the blood circulation through the lung tissue ventilation function and cause multiple systemic diseases especially the respiratory diseases. This study investigated the pathological mechanism of the lungs injury in rats induced by PM2.5 and the effect and mechanism of the Chinese herbal medicine number 2 Feibi Recipe (number 2 FBR) on lungs injury. In this experiment, Wistar rats were used. Lungs injury was induced by PM2.5. Number 2 FBR was used to treat the rats. The result showed that number 2 FBR could improve the lung injury in the rats. Meanwhile, it significantly reduced pathological response and inflammatory mediators including interleukin-6 (IL-6), interleukin-13 (IL-13), interleukin-17 (IL17), monocyte chemotactic protein-1 (MCP-1), and transforming growth factor-*α* (TNF-*α*) and upregulated glutathione peroxidase (GSH-Px) in the PM2.5 induced lung injury in the rats. Collectively, number 2 FBR appears to attenuate the lungs injury in rats induced by PM2.5.

## 1. Introduction

Air pollution is the problem that human beings are facing these days and it is becoming one of the main causes of respiratory diseases. The main cause of it is the development of modern industry and the excessive discharge of automobile exhaust gas.

Atmospheric particulates, the suspended solid and liquid particulates in the atmosphere, are recognized as the most harmful and representative air pollutants. Atmospheric particulate can be divided into 3 types by their size: total aerated particulates (aerodynamic equivalent diameter ≤ 100 *μ*m); respirable particulates (aerodynamic equivalent diameter ≤ 10 *μ*m); and fine particulates (aerodynamic equivalent diameter ≤ 2.5 *μ*m). Particulates with the size of more than 10 *μ*m usually can be blocked by the nose. Particulate with a particle size under 10 *μ*m but larger than 2.5 *μ*m could enter the upper respiratory tract but can be partially excreted out of the body by spitting and sneezing. As for fine particulates with the diameter below 2.5 *μ*m, they can go through the human defense barrier to enter the alveoli and then enter the blood circulation through the lung tissue ventilation function. It can cause multiple systemic diseases, such as asthma, bronchus inflammation, pulmonary fibrosis, chronic obstructive pulmonary disease, and even lung cancer and other respiratory diseases [1].

Since we were not aware if the PM2.5 could cause the disease in rats, we established a model to detect the results. The PM2.5 model rats were established by injecting PM2.5 suspension into the airway. Then we observed the pathological changes of lung tissue, the determination of inflammatory factors, chemokines, oxidative stress markers, alveolar epithelial cell apoptosis, and so on.

Number 2 FBR is a modified formula from FBR, which is a formula according to Professor Ping'an Zhou's more than 50 years' clinical experience. We took the characteristic of the PM2.5 causing the injury into consideration and modified some of the herbs with the function of antifibrosis into the herbs with the function of anti-inflammation, because the FBR is a formula mainly for treating the pulmonary fibrosis. Former research approved that FBR can inhibit the phosphorylation of P38MAPK, decrease the expression of TGF*β*1, downregulate the expression of IL-6 in the lung tissue, and decrease the content of type III collagen and hyaluronic acid in the serum. Therefore, the FBR can inhibit the fibrosis and immunoinflammatory injury [2]. Also the clinical researches have shown that the FBR could improve the life quality of the idiopathic pulmonary fibrosis patients [3, 4].

This research was based on our previous studies. According to the characteristics of PM2.5 induced lungs injury, we modified this formula as number 2 FBR and used it to treat the PM2.5 model rats to observe the difference in sham, model, and number 2 FBR groups.

## 2. Material and Method

### 2.1. Reagents and Materials

Rat IL-17 ELISA Kit (cat number ab119536, lot number GR289617-1), Rat TNF-*α* ELISA Kit (cat number ab100784, lot number GR307317-1), Rat IL-6 ELISA Kit (cat number ab100772, lot number GR308338-1), MCP-1 Antibody (cat number ab25124, lot number GR1152-42), TNF-*α* Antibody (cat number ab6671, lot number GR235155-13), and IL-6 Antibody (cat number ab9324, lot number GR237999-12) were purchased from Abcam. IL-17 Antibody (cat number sc-6077, lot number B0713) was purchased from SantaCruz; IL-13 Antibody (cat. number BA1804-2, lot number 1560109) was purchased from Boster.

### 2.2. Preparation of PM2.5

PM2.5 filters were given as a friendly gift from Environmental Laboratory of Nanjing University. The filters collected the atmospheric particulates of Xianlin Campus of Nanjing University from 2015.5 to 2015.6. The filters were made of Quartz fiber membrane (Whatman). Every filter contained nearly 350 mg PM2.5; 1/2 could be extracted. So, there were nearly 525 mg PM2.5 on 3 filters in total. The filters were cut to less than 1 cm^2^ and put into a centrifugal tube and then poured into deionized water. The Quartz would be dissolved. After that, the centrifugal tube was put into the ultrasonic washer to wash the particulates (40 min, 3 times). The liquid was filtered by eightfold gauze, putting the liquid to the centrifuge, discharging the supernatant and drying it; at last we got 450.2 mg PM2.5. The concentration of added deionized water to the PM2.5 was 10 mg/ml. The main components of it were SO_4_^2−^ (17.2%), NO_3_^−^ (16.9%), NH_4_^−^ (10.5%), and secondary organic aerosol (13.1%).

### 2.3. Preparation of No. 2 FBR

Number 2 FBR was purchased from Beijing Kang Ren Tang, the Pharmaceutical Industry Co. The components of it are number 2 FBR composed of Radix Astragali, Rhodiolae Crenulatae Radix et Rhizoma, Flos Lonicerae Japonicae, Radix Scutellariae, Radix et Rhizoma Salviae Miltiorrhizae, Radix et Rhizoma Glycyrrhizae, and so on. Total net weight is 66 g.

### 2.4. Animal Model and Experiment Design

50 Wistar rats were purchased from Beijing Vital River Laboratory Animal Technology Co., Ltd., weighting 180–200 g. After 3 days of adaptive feeding the model was made. All rats were randomly assigned to sham group (10 rats), model group (20 rats),and number 2 FBR group (20 rats). The model group and number 2 FBR group rats' lungs injury was induced by PM2.5.

The rats were abdominally anesthetized by 10% chloral hydrate (35 mg/kg). The rats were fixed on the inclined plate in supine position with the angle of 45°. The upper teeth and limbs were fixed. Then, pulling out the rat's tongue, stirring the tongue, and putting the neck as extended position, the light was directed through the throat, exposing the glottis. The oral secretions were cleaned up. We inserted number 16 trocar into the rat's trachea and drew out the metal needle, leaving the plastic tube. A cotton wire on the outer edge of the plastic tube was performed to check whether the insertion was successful; if it was, there would be a rhythmic sway of it. The PM2.5 suspension (1 ml/kg·bw) was injected into the trachea through the trocar. The rat plate was then rotated to the left and right uprightly for 20 seconds to make the PM2.5 suspension evenly distributed in the lungs. This operation was done at the 5th, 11th, 17th, and 23rd days. From the 1st day, we gave the saline (10 ml/kg·bw) to the sham group and the model group by gavage once a week for 28 days. Meanwhile, number 2 FBR group rats would take number 2 FBR (10 ml/kg·bw) with the concentration of 0.6787 g/ml once a week for 28 days, and the body surface area between human beings and rats was taken into consideration. The rats were going to be euthanized after 28 days.

### 2.5. Sample Collection

At the end of the 28-day experiment, all the rats were anesthetized by intraperitoneal injection of chloral hydrate and euthanized by exsanguination. Abdominal skin and muscle were cut along the median line, and the abdominal aorta was exposed, drawing and centrifuging the blood with 4°C, 3000 r/min, for 10 min. The separation of supernatant was stored in sterile EP tube. After that, the lungs were separated into 2 parts. The left lobe lung was fixed by polyoxymethylene for HE staining, TUNEL, and immunohistochemical staining, while the right lobe lung was reserved in liquid nitrogen for Western Blot and RT-PCR.

### 2.6. Deaths and Body Weight Changes Measurement

The deaths were recorded, and the body weight changes were recorded on the 1st, 5th, 11th, 17th, 23rd, and 28th day.

### 2.7. General Histological Staining

The left lung was fixed in 10% formalin for more than 24 h and then dehydrated and embedded in paraffin and 5 *μ*m sections were then cut perpendicularly to the vessels of the lung. Follow the steps: the sections were (1) put into xylol I, II, each for 15 mins; (2) put into the ethanol with the concentration of 100%  I, II → 95% I, II → 90% → 80% → 70% → 60% → 50%, each for 2 min, and washed with running water for 5 min; (3) stained with hematoxylin for 15 min, washing the extra hematoxylin; (4) put into 1% hydrochloric acid alcohol for 30 s and washed with running water for 10 min; (5) stained with eosin for 15 min and washed for 1 min, (6) put into the ethanol with the concentration of 50% → 60% → 70% → 80% → 90% → 95% I, II → 100% I, II, each for 2 min; and (7) put into xylol I, II, each for 15 mins. TUNEL was used for apoptosis of alveolar epithelial cells counting. Positive criteria for apoptosis are as follows: brown-black or black particles in the nucleus were apoptotic cells. Each section in 100 and 400 times field of view was randomly taken; in the field, the number of apoptotic cells and cells in total were counted, then calculating the average percentage of the apoptotic cells, which is the average apoptotic index. Positive control: drop through 0.1% ketone-X100, with DNase (1 mg/ml, 10 minutes, room temperature) induced DNA strand breaks. Negative control: 50 uL of the labeled solution (without terminal conversion enzyme) was used to take the place of TUNEL reaction mixture.

### 2.8. Immunohistochemical Staining

The sections were baked at 60°C for 30 minutes, microwave antigen retrieval was performed for 40 minutes, and then endogenous peroxidase was quenched with 3% H_2_O_2_ for 10 minutes at a room temperature and away from light. The sections were incubated with a primary antibody for 20 minutes, and complete washing was performed by PBS, followed by secondary antibody for 20 minutes and washed again. The positive immunostaining in the tissues was visualized by 3,3-diaminobenzidine tetrahydrochloride (DAB), the section was observed for 5 minutes. The sections were stained with Mayer's hematoxylin. The positive expression of the cell would be brown. The section of negative sham group was added to PBS and put into refrigerator at 4°C for a night and washed by PBS. The picture was observed by Olympus microscope (Tokyo, Japan). The data was analyzed by Image Pro Plus 6.0. (Media Cybernetics, Rockville, MD, USA).

### 2.9. ELISA Was Used in Cytokine Measurement

Concentrate IL-6, IL-13, IL17, MCP-1, GSH-Px, iNOS, and TNF-*α* levels in serum were measured by ELISA kits. All procedures were performed in accordance with the manufacturer's instructions.

### 2.10. Western Blot Was Used in Analysis of Protein in Lung Tissue

Levels of MCP-1, TNF-*α*, IL-6, IL-13, and IL-17 expression in full thickness were analyzed. The tissue was homogenized in RIPA lysis buffer. The homogenate was centrifuged at 12000 r/min (the centrifuge from THERMO, legend micro 21 r), for 10 min, and the supernatant was collected. The protein samples were denatured by boiling for 5 minutes in SDS sample buffer (MDL). Afterward, equal quantities of protein were separated with 10% SDS-PAGE (Anngen, Cat. Da2604) and transferred to polyvinylidene difluoride (PVDF). The membranes were blocked with 5% skim milk for 2 h and then incubated with primary antibodies (MCP-1, TNF-*α*, IL-6, IL-13, and IL-17) overnight at 4°C. After exposure to secondary antibody for 60 minutes at 37°C, the membranes were analyzed with ECL reagents. All assays were performed independently and repeated three times.

### 2.11. RT-PCR

The tissue was homogenized in liquid nitrogen. For quantitative RT-PCR, the primers were designed as follows: TNF-*α* 5-GACCAGCCAGGAGGGAGAAC-3 (forward) and 5-TCCGGAGGGAGATGTGTTGC-3 (reverse), IL-6, 5-TTGCCTTCTTGGGACTGATG-3 (forward) and 5-ACTGGTCTGTTGTGGGTGGT-3 (reverse), IL-13, 5-AGCAGCATGGTATGGAGCGT-3 and 5-AAGCCACATCCGAGGCCTTT-3 (reverse), IL-17, 5-TGGTCCTGAAGAGGGAGCCT-3 (forward) and 5-TAGGACGCATGGCGGACAAT-3 (reverse), GAPDH, 5-GTTACCAGGGCTGCCTTCTC-3 (forward) and 5-GGGTTTCCCGTTGATGACC-3 (reverse). It was according to the instruction of SuperScript III RT Reverse Transcription kit (ABI-Invitrogen). All assays were performed in triplicate and independently repeated three times.

### 2.12. Statistical Analysis

All graphing and statistical analyses were performed by using spss17.0. Comparisons among multiple groups were analyzed with one-way ANOVA with a Tukey-Kramer post hoc correction. Single comparisons were made with an unpaired 2-tailed Student's *t*-test. *P* values ≤ 0.05 were considered statistically significant.

## 3. Results

### 3.1. Deaths and Body Weight Changes

**Figure 1:**
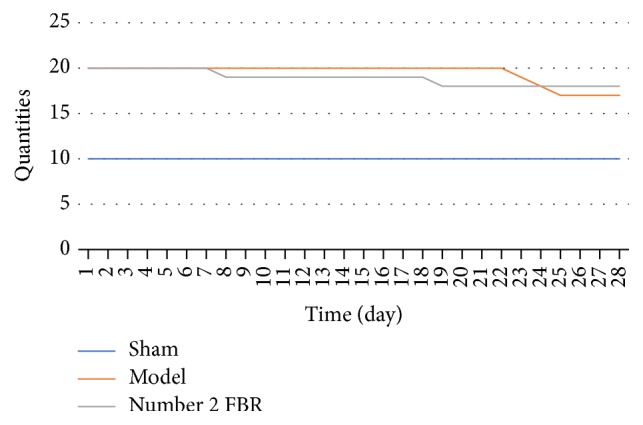
The rats in sham group did not die. Compared with rats in the model group, the number of deaths in number 2 FBR group is less.

The rats in sham group did not die. But in the model group, on the 23rd, 24th, and 25th day, one rat would die. In number 2 FBR group, on the 8th and 19th day, one rat would die. In total, the number of deaths in model group and number 2 FBR group was 3 and 2 (Figure 1).

**Figure 2:**
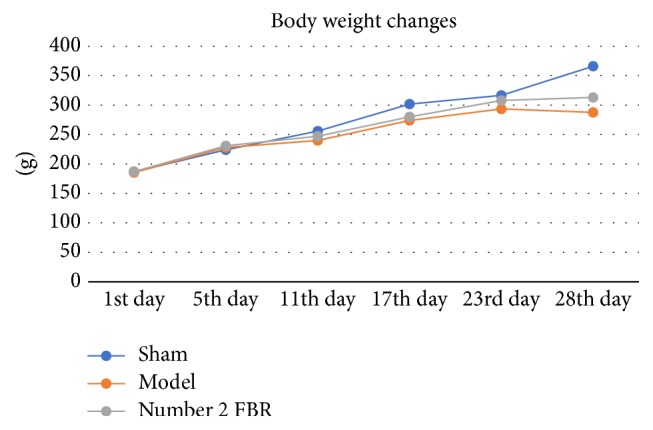
On the 11th, 17th, 23rd, and 28th day, statistic difference was found between sham group and model group (*P* < 0.05). On the 17th and 28th day, statistical difference was found between sham group and number 2 FBR group (*P* < 0.05). On the 23rd and 28th day, statistical difference was found between model group and number 2 FBR group (*P* < 0.05). And the body weight of sham group and number 2 FBR group were increasing through the process of the 28 days' experiment. But it was decreasing in the model group.

The body weight of rats in every group increased in the first 23 days; from 24th day to 28th day, it was still increasing in sham group and number 2 FBR group, but it decreased in model group (see Table 1 and Figure 2).

### 3.2. Number 2 FBR Ameliorates PM2.5 Induced Lungs Injury

**Figure 3:**
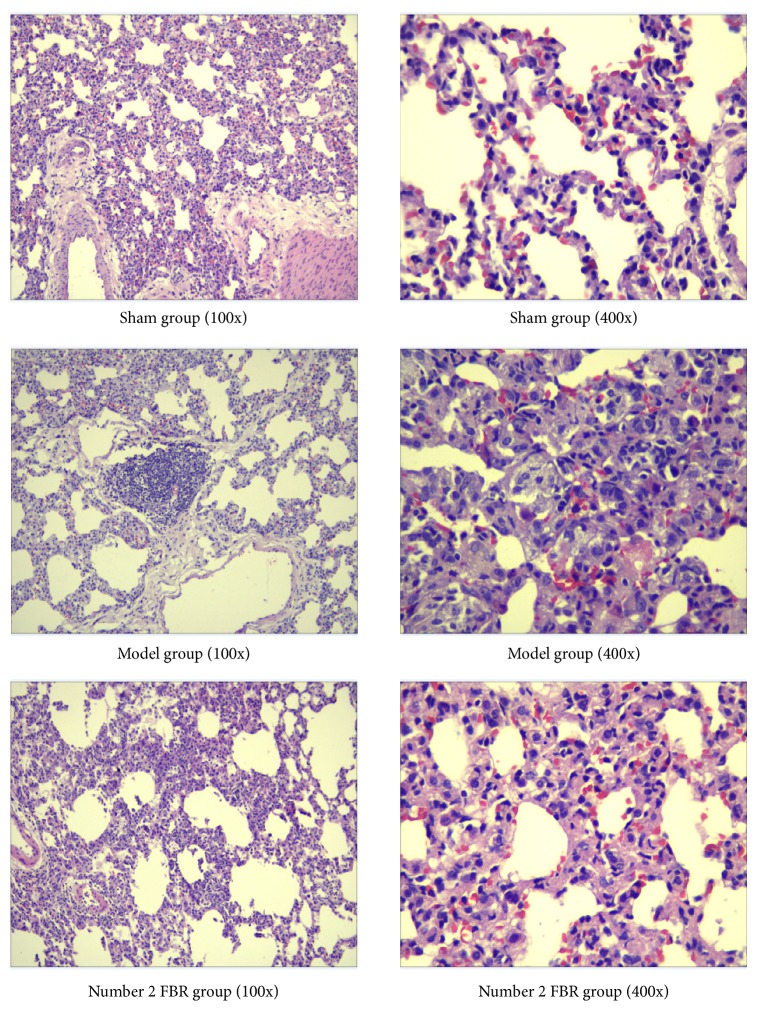
Effect of number 2 FBR on PM2.5-induced pulmonary histopathology in rats. Pulmonary structure and tissue of trachea and pulmonary interstitial inflammatory infiltration in sham group, model group, and number 2 FBR groups. There was less inflammation in filtration with number 2 FBR.

HE staining was performed to evaluate pathological changes in lung tissues. After 4 times of PM2.5 injection to the lungs during the 4 weeks, in the model group, the major pathological injury was inflammatory cell infiltration; in contrast to the sham group, neutrophils and lymphocytes were significantly increased, small number of fibroblasts were showed, and bronchial wall was thickened, accompanied by some bleeding. Meanwhile, the alveolar septum was widened, alveolar cavity was enlarged, and some of the alveolar were ruptured and fused. Compared with model group, number 2 FBR group showed that the inflammatory cell infiltration and the bronchial mucosa injury were improved. The alveolar structure is relatively complete (Figure 3).

### 3.3. Immunohistochemical Staining

**Figure 4:**
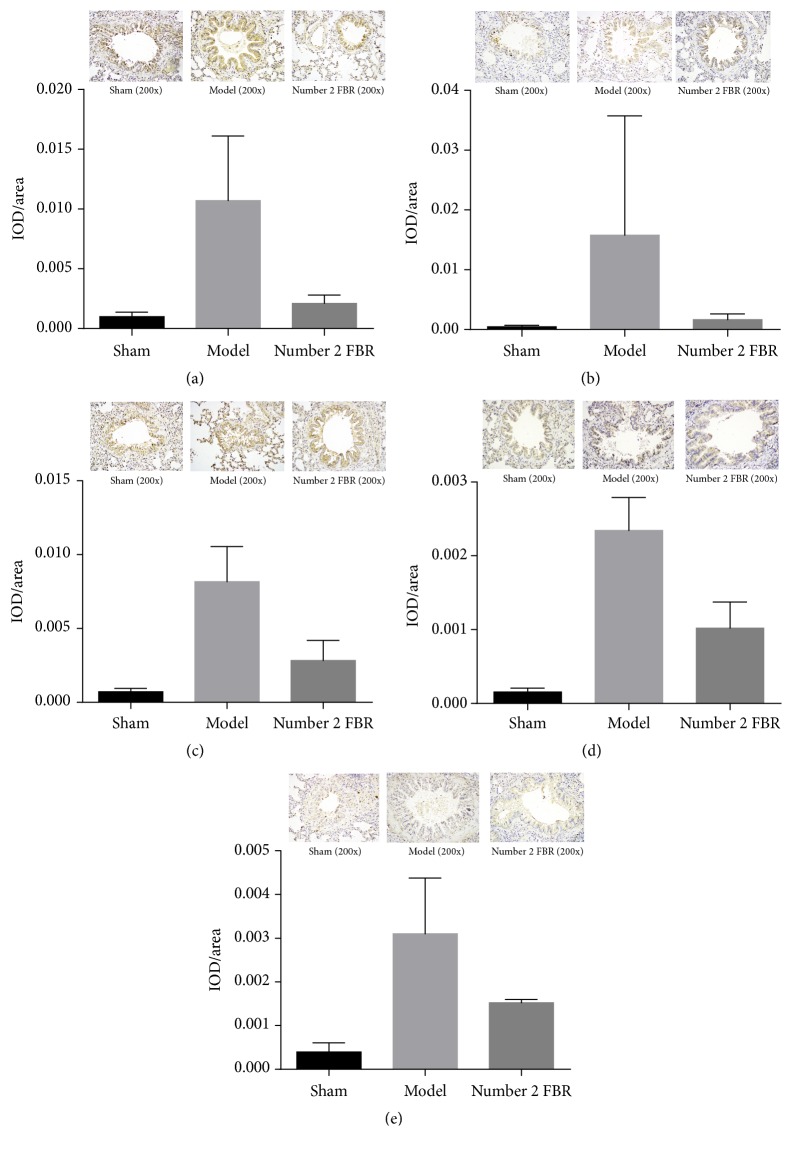
Comparison of the protein of MCP-1 (a), TNF-*α* (b), IL-6 (c), IL-13 (d), and IL-17 (e) in rats lung tissue with the method of immunohistochemical staining. Comparison between sham group and number 2 FBR group with the level of IL-6 (*P* < 0.05); other comparisons of IL-6 and IL-13 level were compared in pairs (*P* < 0.05); comparison of IL-17 level between sham group and model group (*P* < 0.05), comparison of IL-17 level between sham group and number 2 FBR group (*P* > 0.05); comparison of IL-17 level between model group and number 2 FBR group (*P* > 0.05); comparison of MCP-1 level and TNF-*α* between the 3 groups (*P* > 0.05) (Table 2).

Immunohistochemical staining was performed to evaluate protein of MCP-1,TNF-*α*, IL-6, IL-13, and IL-17 deposition in lung tissues. IOD/Area was the parameter to be measured by the Imagine Pro Plus 16.0. From the pictures, we could find that there were more brown spots in the model group, and the pictures of number 2 FBR group were cleaner than the model group, so it is shown that the PM2.5 could elevate the inflammation, and number 2 FBR may inhibit it (Figure 4).

From the analyzed results, we could find from the levels of IL-6 and IL-13 that there was no significant difference between sham group and number 2 FBR group when compared with the level of IL-6 (*P* < 0.05), but there were significant differences in the other comparisons when the levels of IL-6 and IL-13 were compared in pairs (*P* < 0.05). As for the level of IL-17, there was statistical difference between sham group and model group (*P* < 0.05), but there was no statistical difference between sham group and number 2 FBR group; also, there was no significant different between Model group and number 2 FBR group (*P* > 0.05). Furthermore, there was no significant difference in the level of MCP-1 and TNF-*α* between the 3 groups (*P* > 0.05), but in these groups, number 2 FBR still had the trend to decrease the inflammatory markers (see Table 2 and Figure 4).

### 3.4. Effect of Number 2 FBR on Levels of IL-6, IL-13, IL-17, MCP-1, TNF-*α* GSH-Px, and iNOS in Serum

The cytokines were measured by ELISA kits. Compared with sham group, the levels of IL-6, IL-13, IL-17, MCP-1, and TNF-*α* were greater in model group and number 2 FBR group with statistical difference (*P* < 0.05). Compared with number 2 FBR group, the level of IL-6, IL-13, IL-17, MCP-1, and TNF-*α* in the model group was greater with the statistical difference (*P* < 0.05). Compared with model group and number 2 FBR group, the level of GSH-Px in the sham group was greater and significant difference was also found (*P* < 0.05), and the level of that in number 2 FBR group was greater than that in the model group (*P* < 0.05). There was no statistical significance comparing the level of iNOS in 3 groups (*P* > 0.05) (Table 3).

### 3.5. Effect of Number 2 FBR on Levels of the Protein of MCP-1, TNF-*α*, IL-6, IL-13, and IL-17, in Lung Tissue

Western blotting was used. There was no statistical significance comparing the level of MCP-1, TNF-*α*, IL-6, IL-13, and IL-17 expression in 3 groups (*P* > 0.05). But as for IL-13 in the model group, it was significantly greater compared with sham group and number 2 FBR group (*P* < 0.05). There was no statistical significance comparing the level of IL-13 between sham group and number 2 FBR group (*P* > 0.05) (Table 4).

### 3.6. Effect of Number 2 FBR on Levels of the mRNA of IL-6, IL-13, IL-17, and TNF-*α* in Rats' Lung Tissue

GAPDH was used as the reference, and the mRNA level was quantified by 2^−ΔΔCt  ^ method.

The level of IL-6 in the model group was greater than that in the sham group and number 2 FBR group (*P* < 0.05). As for the level of IL-6, there was no statistical significance between sham group and number 2 FBR group (*P* > 0.05). Furthermore, there was no significant difference in the level of IL-13, IL-17, and TNF-*α* between the 3 groups (*P* > 0.05) (Table 5).

## 4. Discussion

With the rapid development of modern world, the environment we rely on has been seriously damaged, and the air pollution is one of the causes. The main contaminant of the air is PM (Particulate Matter). It is composed not only from inorganic matters, such as sulfate and nitrate, but also from organic matters such as organic aerosol particles. Also, bacteria, mold, trace metal, and carbon elements are also included [5, 6]. The most harmful particles to human health are PM10 (aerodynamic equivalent diameter ≤ 100 *μ*m) and PM2.5 (aerodynamic equivalent diameter ≤ 2.5 *μ*m).

Studies [7, 8] have shown that the concentration of PM2.5 was associated with the morbidity and mortality of respiratory and cardiovascular system disease. Also, PM2.5-induced respiratory injury is a complex pathological process, the mechanism of it is that, firstly, PM2.5 enters lung tissue and interacts with macrophages and pulmonary epithelial cells in the alveoli; toxic component would be adsorbed that can cause the release of inflammatory factors and changes of biochemical components. Meanwhile, it could induce local inflammation and lead to pulmonary fibrosis; secondly, the fine particles have the function of free radical activity, also the metal and organic components which it contains can stimulate the macrophages to generate free radicals, and oxygen free radicals can activate nuclear regulatory proteins that can cause many cytokines, resulting in immune disorders. Thirdly, some of the components can be regarded as antigen and hapten, which can activate the body immune response; oxygen is consumed in the process of macrophage phagocytosis, which generates many extracellular reactive oxygen species (ROS), and it can directly damage the genetic material, leading to the mutation or activation of oncogene and inactivation of tumor suppressor gene [9].

Number 2 FBR is composed of Radix Astragali, Rhodiolae Crenulatae Radix et Rhizoma, Flos Lonicerae Japonicae, Radix Scutellariae, Radix et Rhizoma Salviae Miltiorrhizae, Radix et Rhizoma Glycyrrhizae, and so on.

Pharmacological studies have shown that [10] Radix Astragali contains astragalus saponins and polysaccharides. Astragalus saponins and polysaccharides have the function of immunoregulation. Astragalus polysaccharide can protect the rat's lung tissue from pulmonary fibrosis. Meanwhile, astragaloside, amino acids, and selenium have the function of antiaging, antifree radical damage, antilipid peroxidation, and anti-inflammatory effects.

Rhodiolae Crenulatae Radix et Rhizoma can improve the activities of SOD and CAT GSH-Px in the liver tissue, and also it could decrease the MDA in the plasma and the lipofuscin in the liver [11].

Flos Lonicerae Japonicae contains flavonoids and chlorogenic acid which has the effects of broad-spectrum antibacteria and antivirus; also, the components have anti-inflammatory, antipyretic, and antiendotoxin functions. Moreover, they can regulate the immunity, promote phagocytic function of leukocytes, reduce the inflammatory cells in lung tissue, and reduce the hydroxyproline content [12].

The active ingredients of the Radix Scutellariae, such as Baicalin, Baicalein, and Wogonin, have the function of antivirus, antibacterial, and antioxidation. Researches have shown that Baicalin could directly remove free radicals, superoxide anion, and other oxygen free radicals and inhibit the activity of xanthine oxidase, so it is a good antioxidant [13].

The main active ingredients of Radix et Rhizoma Salviae Miltiorrhizae could improve the microcirculation and inhibit the platelet aggregation and thrombosis; also, it has antibacterial and anti-inflammatory functions and improves the central sedation. Tanshinone, tanshinol, and salvianolic acid were proved to be effective antioxidants in vitro and in vivo. The antioxidant activity of salvianolic acid was the strongest; the antioxidant activity and free radical scavenging effects of it were stronger than VitC [14].

Radix et Rhizoma Glycyrrhizae mainly contains glycyrrhizin, flavonoids, and alkaloids, with broad-spectrum antibacterial and antiviral effects. Also, the components have the function of anti-inflammation and antiallergy. Meanwhile, it can relieve cough and asthma to protect the throat and tracheal mucosa. Moreover, they can protect the liver function and have the similar effect like adrenal cortical hormone [15].

In this study, Wistar rats were used, and the suspension of PM2.5 particles was injected into the lungs of the rats as the model. The rats were treated with number 2 FBR; after the treatment we detected IL-6, IL-13, IL-17, MCP-1, iNOS, and GSH-PX in the serum and the lung tissue to explore the molecular mechanism and signal transduction pathways of lung injury induced by PM2.5 and the effect of number 2 FBR on it at the gene and protein level.

IL-6 is a multifunctional inflammatory cytokine and a key component of the inflammatory mediator network; it plays an important role in the inflammatory response. As anti-inflammatory cytokines and long-term cytokines, it can balance the damage of proinflammatory cytokines. Due to its two-way function of producing inflammation and anti-inflammation, its role is related to the content of the tissue. The normal level of it can be beneficial, but too much of it can cause a series of inflammatory lesions [16]. IL-13 can enhance mucus secretion by promoting goblet cell hyperplasia [17] and improve mucus secretion to thicken the wall of airway [18]. Also, it could increase the expression of vascular cell adhesion molecule 1 (VCAM-1) and promote the aggregation eosinophils and T cells in the bronchial mucosa [19]. The overexpression of IL-13 in the airway also shows the excessive secretion of mucus and inflammation and eventually leads to pulmonary fibrosis.

IL-17 is a proinflammatory cytokine that binds to IL-17 receptors to induce the secretion of inflammatory cytokines and chemokines such as granulocyte colony stimulating factor (G-CSF), macrophage inflammatory protein-2 (MIP-2), monocyte chemoattractant protein (MCP), IL-6, IL-8, CXCL-1, CXCL-10, prostaglandin E2, NO, matrix metalloproteinases (MMP), and acute phase proteins. IL-17 promotes the secretion of these factors and then attracts the neutrophils into the inflammatory site [20].

MCP-1 also can raise lymphocytes, eosinophils, and macrophages to the alveolar septum and alveolar cavities. These cells can secrete growth factors such as transforming general factor-*β*1 (TGF-*β*1) and thus increase the synthesis of collagen fibers and fibroblast proliferation that initiates the process of pulmonary fibrosis resulting in irreversible damage [21].

GSH-Px is an important peroxidative enzyme which is widely found in the body and specifically catalyzes glutathione (GSH) to the reduction of peroxides. This reaction can clean the oxide metabolites and block lipid peroxidation chain reaction, which plays a role of protecting the cell membrane structure and function.

iNOS produces oxygen free radicals and nitroperoxides under certain pathological conditions. These products can cause severe cell damage. So, the test of activity of iNOS can reflect the degree of lipid oxidation, indirectly reflecting the level of free radicals.

The activity of GSH-Px reflects the ability of the body to scavenge oxygen free radicals, and the activity of iNOS indirectly reflects the severity of the cell attacked by the free radical.

Tumor necrosis factor-*α* (TNF-*α*) is a multiactive cytokine secreted by endotoxin-activated macrophages and lymphocytes. It is the most powerful cytokine for antitumor and anti-inflammation. It is related to tumor, infection, fever, endotoxic shock, autoimmune diseases, and transplant rejection. The more severe the condition is, the higher the TNF-*α* value would be [22]. TNF-*α* has dual biological functions. On the one hand, it is an important medium of the protective immunity; on the other hand, it involves in the body's immune pathological damage; it plays an important role in the immune disease. Normal levels of TNF-*α* can regulate the immune response, work as anti-infection, promote tissue repair, and cause tumor cell apoptosis. But too much of it will cause immune imbalance and lead to pathological damage. TNF-*α* also stimulates the release of other cytokines such as IL-4 and IL-6, expands the biological effects of them, and mediates the posttraumatic cell inflammatory responses and causes multiple organ tissue damage.

The results of this study show that the death rate of the model group increased in the process, and the weight increasing is lower than the sham group. And the death rate of number 2 FBR group is lower than that of the model group and the weight increasing is higher than that of the model group. That indicates that the infusion of PM2.5 contributed to the increasing death rate and decreasing weight gain, while Chinese medicine can improve this situation.

In the HE staining, we could see the infiltration of inflammatory cells obviously, and some of fibroblasts were observed. The bronchial mucosa was partially damaged and the bronchial wall was thickened and widened, alveolar cavity was enlarged, partial alveolar was ruptured and fused, and there were secretions in the cavity. We could see they are mainly mononuclear cells and neutrophils. Meanwhile, the lymphocyte hyperplasia and alveolar consolidation could be found. Pulmonary capillaries were with blood and inflammatory exudation; capillary wall was thickened. As we could see in number 2 FBR group, the inflammatory, bronchial mucosal injury, and cavity secretion were improved. Alveolar and capillary structures were nearly complete, seldom blood was showed. So, the PM2.5 could damage the lung tissue of rats, and lead to the airway injury in rats, that could be used in the research. As a result, the number 2 FBR could improve the PM2.5 induced lung injury.

From the result of ELISA, the levels of IL-6, IL-13, IL-17, MCP-1, and TNF-*α* in the serum of the model group were higher than those in the sham group, and these in number 2 FBR group were decreased compared with these in the model group. The expression of GSH-Px in the serum in the model group was higher than that in the sham group, but that among the three groups showed no significant difference. It suggested that the suspension of PM2.5 in the airway can promote the release of inflammatory factors, amplify the inflammatory reaction, and increase the synthesis of collagen fibers and the proliferation of fibroblasts. So it initiates the process of pulmonary fibrosis. Moreover, it could reduce the catalytic effect of GSH on of the reduction reaction of peroxide which helped to reduce the body's ability to remove peroxide metabolites. Inversely, the lipid peroxidation relatively enhanced the destruction of the cell membrane structure. Meanwhile, cytokines in serum increased causing multiple organ damage. Number 2 FBR has effect on reducing the damage of inflammatory factors and inhibiting the inflammatory reaction, thus inhibiting the deposition of collagen and the occurrence of fibrosis. For all that, number 2 FBR could protect the cell membrane structure by enhancing catalytic effect of GSH on the reduction reaction of peroxide and scavenging free radicals.

Western blot was used to detect the expression of the proteins in lung tissue of rats. Only the comparison of IL-13 had the statistical difference between 3 groups. The IL-13 in the model group was higher than that in the sham group and number 2 FBR group, indicating that the infusion of PM2.5 in the airway could increase the inflammation and promote the excessive secretion of mucus and lead to significant pulmonary fibrosis. Number 2 FBR could inhibit the protein expression IL-13, thereby inhibiting the inflammation and the excessive secretion of mucus. There was no statistical difference in the expression of MCP-1, TNF-*α*, IL-6, and IL-17 between the model group and sham group. But we could see that the trend of these in the model group was elevated, indicating the target that PM2.5 causes lung tissue injury in the protein translation stage. The trend of the expression of TNF-*α* and IL-6 in number 2 FBR group was lower than that in the model group, which indicated that the target of these 2 inflammatory factors was reduced in protein translation stage. If we prolong the process of number 2 FBR treatment, the differences could be more obvious.

The result of immunohistochemical staining was almost the same with western blot; because the immunohistochemical staining was usually used to detect the position of the protein, the western blot was used to detect the quantity. So, the result of western blot was more accurate. As we could see from the pictures that compared with the sham group there were more brown spots in the picture of the model group, and in the bronchial cavity it is not clean, in number 2 FBR group we could find less brown spots than the model group, since number FBR may inhibit the inflammation.

The results of RT-PCR showed that only the level of IL-6 was significantly different in the 3 groups, that in the model group was higher than that in the sham group and No. 2 FBR group. The results showed that the expression of IL-6 mRNA in the model group was upregulated, in the gene level the expression of IL-6 could be inhibited by No. 2 FBR.

## 5. Conclusion

We noted that PM2.5 could cause the lungs injury, stimulate tissue inflammatory factors, reduce the catalytic effect of GSH on of the reduction reaction of peroxide and increase the synthesis of collagen fibers. Meanwhile, number 2 Feibi recipe could reduce release of inflammatory cytokines and enhance catalytic effect of GSH on the reduction reaction of peroxide and scavenging free radicals and alleviated injury in PM2.5 induced rats.

## Figures and Tables

**Table 1:** Comparison of body weight changes in 3 groups.

Group	1st day	5th day	11th day	17th day	23rd day	28th day
Sham	186.69 ± 7.61	224.16 ± 7.41	255.76 ± 10.10	301.57 ± 24.39	316.59 ± 23.69	366.04 ± 19.44
Model	185.48 ± 5.25	227.93 ± 6.51	240.11 ± 14.40^*∗*^	274.09 ± 19.20^*∗*^	293.57 ± 21.85^*∗*^	287.68 ± 22.16^*∗*^
Number 2 FBR	187.03 ± 5.92	231.07 ± 8.22	247.18 ± 10.26	279.95 ± 12.31^*∗*^	307.84 ± 15.31^△^	313.10 ± 15.21^*∗*△^

^*∗*^Compared with sham group *P* < 0.05; ^△^compared with model group *P* < 0.05.

**Table 2:** Comparison of the protein of MCP-1, TNF-*α*, IL-6, IL-13, and IL-17 in rats lung tissue with the method of immunohistochemical staining.

Group	MCP-1	TNF-*α*	IL-6	IL-13	IL-17
Sham	0.00998 ± 0.00038	0.00046 ± 0.00026	0.00072 ± 0.00022	0.00016 ± 0.00005	0.00040 ± 0.00021
Model	0.01070 ± 0.00541	0.01580 ± 0.01995	0.00817 ± 0.00237^*∗*^	0.00234 ± 0.00045^*∗*^	0.00310 ± 0.00128^*∗*^
Numner 2 FBR	0.00210 ± 0.00070	0.00165 ± 0.00097	0.00283 ± 0.00136^△^	0.00102 ± 0.00035^*∗*△^	0.00152 ± 0.00007

^*∗*^Compared with sham group *P* < 0.05; ^△^compared with model group *P* < 0.05.

**Table 3:** Comparison of cytokines in serum of rats in the 3 groups.

Group	IL-6	IL-13	IL-17	MCP-1	GSH-Px	iNOS	TNF-*α*
Sham	100.66 ± 15.29	20.89 ± 3.41	27.61 ± 2.35	513.47 ± 104.37	174.25 ± 30.38	11.55 ± 1.83	189.10 ± 15.06
Model	177.73 ± 25.80^*∗*^	36.84 ± 5.88^*∗*^	31.34 ± 4.25^*∗*^	783.44 ± 126.36^*∗*^	100.32 ± 15.89^*∗*^	10.91 ± 1.30	353.21 ± 49.14^*∗*^
Number 2 FBR	131.87 ± 17.77^*∗*△^	30.05 ± 5.12^*∗*△^	30.72 ± 3.99^*∗*△^	594.32 ± 98.53^*∗*△^	131.71 ± 18.33^*∗*△^	11.24 ± 1.16	283.16 ± 34.79^*∗*△^

^*∗*^Compared with sham group *P* < 0.05; ^△^compared with model group *P* < 0.05.

**Table 4:** Comparison of the protein of MCP-1, TNF-*α*, IL-6, IL-13, and IL-17 in rats lung tissue.

Group	MCP-1	TNF-*α*	IL-6	IL-13	IL-17
Sham	0.18 ± 0.10	0.31 ± 0.24	0.17 ± 0.08	0.29 ± 0.08	0.19 ± 0.08
Model	0.24 ± 0.14	0.53 ± 0.14	0.45 ± 0.14	0.71 ± 0.06^*∗*^	0.33 ± 0.09
Number 2 FBR	0.24 ± 0.06	0.31 ± 0.12	0.33 ± 0.26	0.29 ± 0.08^△^	0.36 ± 0.20

^*∗*^Compared with sham group *P* < 0.05; ^△^compared with model group *P* < 0.05.

**Table 5:** Comparison of the genes IL-6, IL-13, IL-17, and TNF-*α* in rats lung tissue.

Group	IL-6	IL-13	IL-17	TNF-*α*
Sham	0.26 ± 0.29	0.85 ± 1.43	0.42 ± 0.86	11.15 ± 5.33
Model	2.85 ± 7.40^*∗*^	1.01 ± 3.01	0.28 ± 0.97	9.23 ± 3.88
Number 2 FBR	0.81 ± 1.03^△^	1.05 ± 2.15	0.47 ± 0.68	11.65 ± 7.30

^*∗*^Compared with sham group *P* < 0.05; ^△^compared with model group *P* < 0.05.
